# PyroTyping, a novel pyrosequencing-based assay for *Mycobacterium tuberculosis* genotyping

**DOI:** 10.1038/s41598-017-06760-5

**Published:** 2017-07-28

**Authors:** B. Molina-Moya, A. Lacoma, N. García-Sierra, S. Blanco, L. Haba, S. Samper, J. Ruiz-Manzano, C. Prat, C. Arnold, J. Domínguez

**Affiliations:** 1 Servei de Microbiologia, Hospital Universitari Germans Trias i Pujol, Institut d’Investigació Germans Trias i Pujol, Universitat Autònoma de Barcelona, Carretera del Canyet s/n, 08916 Badalona, Spain; 2 Servei de Pneumologia, Hospital Universitari Germans Trias i Pujol, Institut d’Investigació Germans Trias i Pujol, Universitat Autònoma de Barcelona, Carretera del Canyet s/n, 08916 Badalona, Spain; 3 CIBER Enfermedades Respiratorias (CIBERES), Instituto de Salud Carlos III, Madrid, 28029 Spain; 4 Instituto Aragonés de Ciencias de la Salud, Zaragoza, 50009 Spain; 5 Fundación Instituto de Investigación Sanitaria de Aragón, Zaragoza, 50009 Spain; 6 Genomic Services and Development Unit, Public Health England, 61 Colindale Avenue, London, United Kingdom

## Abstract

We developed a novel method, PyroTyping, for discrimination of *Mycobacterium tuberculosis* isolates combining pyrosequencing and IS*6110* polymorphism. A total of 100 isolates were analysed with IS*6110*-restriction fragment length polymorphism (RFLP), spoligotyping, mycobacterial interspersed repetitive units – variable number tandem repeats (MIRU-VNTR), and PyroTyping. PyroTyping results regarding clustering or discrimination of the isolates were highly concordant with the other typing methods performed. PyroTyping is more rapid than RFLP and presents the same discriminatory power, thus, it may be useful for taking timely decisions for tuberculosis control.

## Introduction

Tuberculosis (TB) is one of the leading causes of death among curable infectious diseases, and worldwide spread of *Mycobacterium tuberculosis* isolates, the causative agent, poses a threat to the global control of TB^1^. In order to improve TB control, it is important to track the spread of *M. tuberculosis* isolates, identify index cases, and detect outbreaks. For these purposes, several genotyping methods have been developed^2^. Some of these methods are based on the polymorphism of copy number and location of the insertion sequence (IS *6110*. The most used IS*6110*-based genotyping method is restriction fragment length polymorphism (RFLP)^3^. However, RFLP is laborious and time-consuming, thus, the time until results are available may be too long for decision-making. Another extensively used genotyping method is spoligotyping^4^, based on the polymorphism of the clustered regularly interspaced short palindromic repeats (CRISPR) locus in the *M. tuberculosis* genome. Although spoligotyping is more rapid and easy to perform than RFLP, it presents a lower discriminatory power^2^. Finally, the molecular typing method considered as gold standard is the mycobacterial interspersed repetitive units - variable number of tandem repeats (MIRU-VNTR), based on the polymorphism of number of the repeats in 24 loci in the *M. tuberculosis* genome^2^. However, the different protocols for this method are either expensive (fluorescence-based sequencing) or time consuming (gel electrophoresis). Hence, new rapid, simple, and discriminatory methods for molecular epidemiology studies could be useful.

The aim of the present study was to develop a novel molecular method based on PCR amplification and pyrosequencing (PyroTyping) for discrimination of isolates based on the polymorphism of the IS*6110* insertion site. In addition, PyroTyping results regarding clustering or discrimination of the isolates were compared with RFLP, spoligotyping, and 24-loci MIRU-VNTR results.

**Figure 1:**
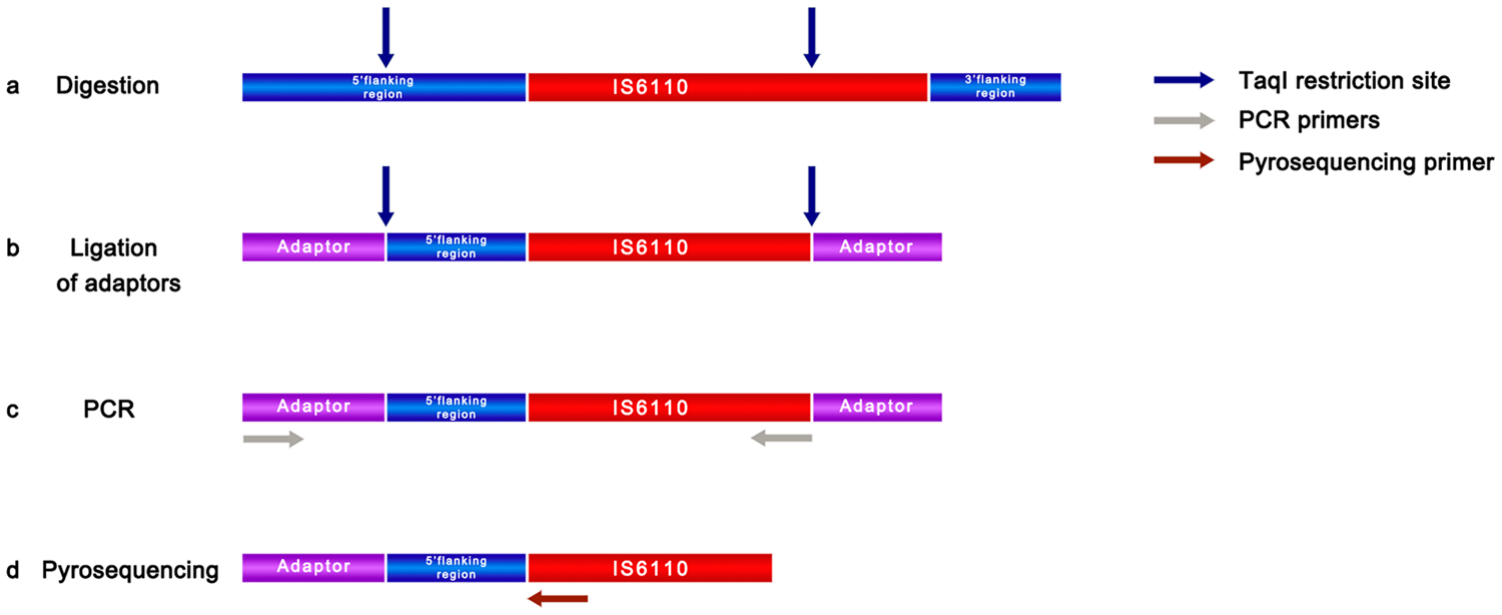
Schematic diagram of the PyroTyping assay. Genomic DNA of *M. tuberculosis* isolates grown in Löwenstein-Jensen solid medium was extracted by the cetyltrimethylammonium bromide protocol. After, DNA was digested with Tru1I (MseI) and *Taq*I restriction enzymes (note that the arrows no dot represent the exact restriction sites) (**a**); adaptors were ligated to the *Taq*I restricted fragments (**b**); the fragments were amplified by PCR using a biotinylated primer complementary to the adaptor and a primer complementary to the IS*6110* (**c**); finally, the amplification product was subjected to pyrosequencing of the 5′ IS*6110*-flanking regions by using a primer complementary to the 5′ end of the IS*6110* (**d**).

The PyroTyping assay (Fig. 1) consists of digestion of the *M. tuberculosis* genomic DNA with *Taq*I restriction enzyme, which cuts on a target located within the IS*6110* and in a target located 5′ to the IS*6110*, dependent on insertion point; ligation of adaptors; touchdown PCR for amplification of the 5′ IS*6110*-flanking region of all the IS*6110* copies present in the genome; and simultaneous pyrosequencing of the amplified fragments. When two isolates share the same RFLP pattern, the IS*6110* copies are located in the same position in the genome, and thus, the sequence of the IS*6110*-5′ flanking regions would be identical. In this case, pyrosequencing profiles would also be identical. On the contrary, when two isolates exhibit different RFLP patterns, pyrosequencing profiles would also be different. The PyroTyping assay was shown to be reproducible in the three independent reactions performed using DNA extracted from the *M. tuberculosis* reference strain H37Rv. In addition, the limit of detection was set at 100 ng of DNA.

The PyroTyping assay was performed in a set of 100 *M. tuberculosis* isolates: 94 isolates corresponded to 29 molecular epidemiology case studies that were part of standard investigations of contact tracing or suspected cases of laboratory cross-contamination (Table 1), and the remaining six isolates were multidrug-resistant (MDR) isolates from patients from different countries (two patients from Morocco, two from Romania, one from Colombia, and one from Spain) for which there was not known or suspected link. RFLP and spoligotyping were performed for all the isolates, and MIRU-VNTR was performed for the 86 isolates for which DNA was available (78 isolates corresponding to 24 case studies and the six MDR isolates).

**Table 1:** Case studies of molecular epidemiology investigations.

Type of investigation	No. of case studies (no. of isolates)	No. of patients in each case study (no. of patient isolates clustered by RFLP)
Contact tracing	22 (72)	
Family members	9 (23)	3 (3), 2 (2), 3 (3), 2 (2^a^), 2 (2), 4 (4), 3 (3), 2 (2), 2 (2)
Spatio-temporal proximity	5 (32)	15 (4, 2, 2, 2), 5 (5), 2 (0), 8 (8^b^), 2 (2^b^)
Cohabitants	3 (7)	3 (2), 2 (2), 2 (0)
Workplace	4 (8)	2 (0), 2 (0), 2 (2), 2 (2)
Friends	1 (2)	2 (0)
Laboratory cross-contamination	7 (22)	3 (3), 3 (2), 6 (4), 2 (2), 3 (2), 3 (3), 2 (2)

^a^One of the isolates within the cluster presented two additional bands in RFLP.

^b^One of the isolates within the cluster presented an additional band in RFLP.

RFLP: restriction fragment length polymorphism.

**Figure 2:**
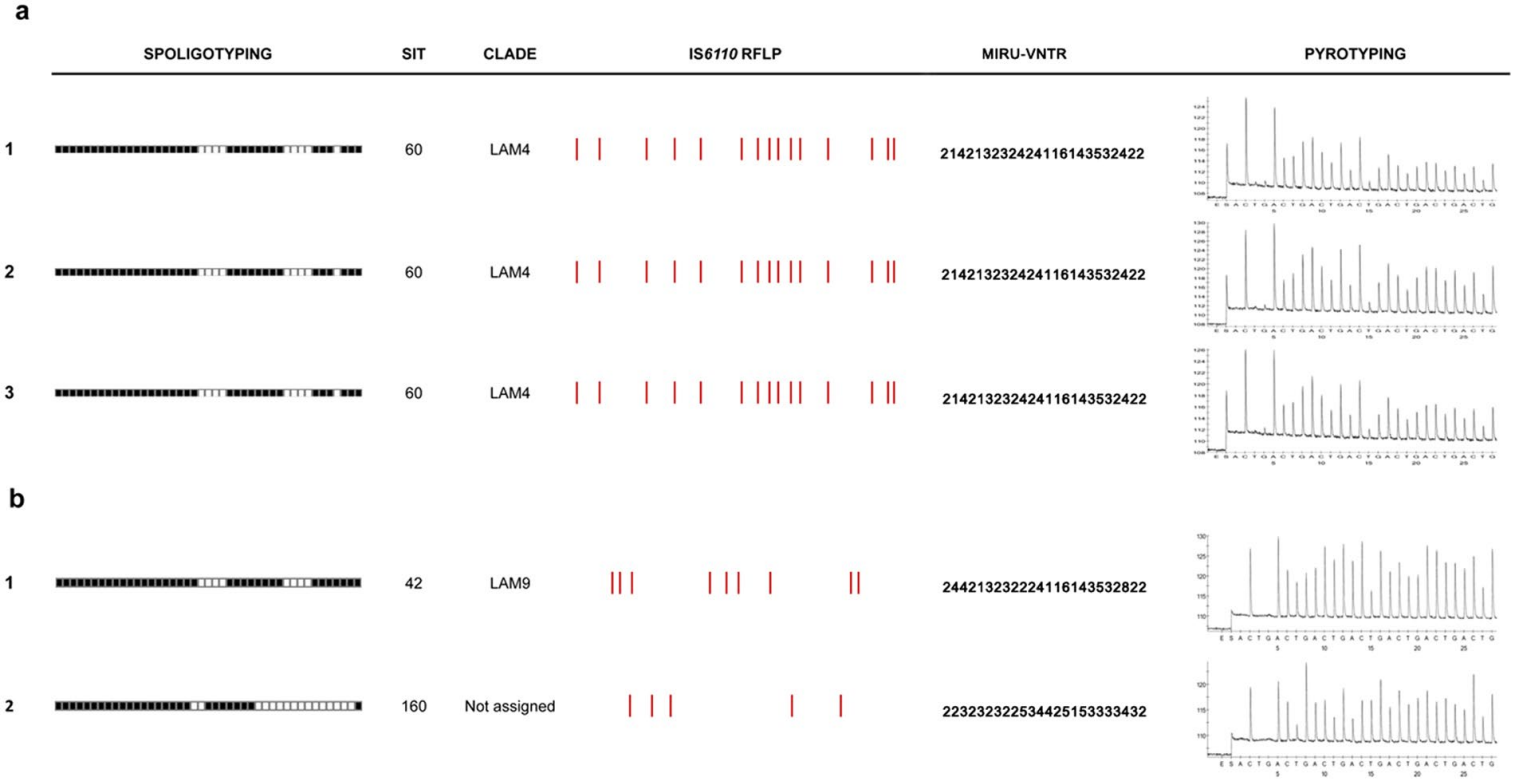
Examples of spoligotyping, IS*6110*-RFLP, 24-loci MIRU-VNTR, and PyroTyping results for some of the *M. tuberculosis* isolates included in this study. (**a**) Results for isolates sharing the same spoligotyping, IS*6110*-RFLP, 24-loci MIRU-VNTR, and PyroTyping profiles. (**b**) Results for isolates showing different spoligotyping, IS*6110*-RFLP, 24-loci MIRU-VNTR, and PyroTyping profiles. RFLP: restriction fragment length polymorphism. MIRU-VNTR: mycobacterial interspersed repetitive units - variable number of tandem repeats.

Results between RFLP, spoligotyping, and PyroTyping for the 100 *M. tuberculosis* isolates were concordant: the three methods agreed on clustering for 74 isolates (Fig. 2a), and on discrimination for 26 isolates (Fig. 2b) (Table 1). Results of MIRU-VNTR were concordant with the other methods except in three cases (Table 2). In addition, slight variations in the RFLP and/or the MIRU-VNTR patterns were detected in three case studies (Table 2). Spoligotyping and MIRU-VNTR patterns are available as Supplemental Material.

**Table 2:** Clustering and discrimination results of RFLP, spoligotyping, MIRU-VNTR, and PyroTyping for the isolates showing variations in the RFLP and/or the MIRU-VNTR patterns.

Case study	No. of isolates	RFLP result	Spoligotyping result	MIRU-VNTR result	PyroTyping result
7	4	Clustered	Clustered	Clustered	Clustered
		(5 bands)	(pattern not described in SITVITWEB)	(one isolate with 1 different locus)	
12	2	Clustered	Clustered	Clustered	Clustered
		(15 bands and 17 bands)	(pattern not described in SITVITWEB)		
17	5	Clustered	Clustered	Clustered	Clustered
		(9 bands)	(SIT53, T1)	(1 different locus^a^)	
19	2	Discriminated	Discriminated	Clustered	Discriminated
		(8 bands and 10 bands)	(SIT58, T5-Madrid2; SIT33, LAM3)		
28	8	Clustered	Clustered	Discriminated	Clustered
		(seven isolates with 10 bands, one isolate with 11 bands)	(pattern not described in SITVITWEB)	(4 different loci)	
29	2	Clustered	Clustered	Discriminated	Clustered
		(9 bands and 10 bands)	(SIT53, T1)	(7 different loci)	

^a^One isolate had 3 repeats, two isolates had 6 repeats, and two isolates had more than 15 repeats.

RFLP: restriction fragment length polymorphism.

MIRU-VNTR: mycobacterial interspersed repetitive units - variable number of tandem repeats.

Among the 100 isolates, 11 isolates had a low IS*6110* copy number (considered as six or fewer bands in the RFLP pattern^2^). These 11 isolates corresponded to four case studies (case studies 2, 7, 14, and 26) involving 3, 5, 2 and 2 isolates, respectively (one isolate had a high IS*6110* copy number). The three isolates of case study 2 were clustered by RFLP (one band), spoligotyping (SIT334, T1), and PyroTyping, but MIRU-VNTR could not be performed. Regarding the five isolates of case study 7, four isolates were those described in Table 2, whereas the other isolate presented a different RFLP pattern (three bands), spoligotyping (SIT326, AFRI_1), MIRU-VNTR, and PyroTyping profile. The two isolates of case study 26 were clustered by RFLP (three bands), spoligotyping (SIT41, LAM7-TUR), MIRU-VNTR, and PyroTyping. The two isolates of case study 14 presented different RFLP patterns (five and nine bands, respectively), spoligotyping (SIT42, LAM9; SIT160, clade not assigned), MIRU-VNTR, and PyroTyping profiles.

Genotyping of *M. tuberculosis* isolates is a valuable tool for TB control and has improved knowledge about TB epidemiology^2, 5^. The most widely used method based on IS*6110* has been RFLP, but it presents limitations for taking rapid decisions for TB control. To overcome these disadvantages, different rapid genotyping assays based on PCR and the polymorphism of IS*6110* have been developed. Some of these methods are based on DNA digestion with restriction enzymes, ligation of adaptors, PCR amplification, and analysis of the amplified fragments. The most used and improved assays have been ligation-mediated PCR^6, 7^ and mixed-linker PCR^8, 9^. Most of these methods perform a gel electrophoresis after PCR, and the fingerprint patterns obtained may display a low number of bands, which limits the level of discrimination that may be achieved. In contrast, in the PyroTyping assay we carried out a touchdown PCR for specific and efficient amplification of the IS*6110* 5′-flanking regions, followed by pyrosequencing, which yields a pyrogram with a variable number of nucleotide peaks, allowing better discrimination compared to the limited number of bands of a gel electrophoresis. In fact, there was complete concordance between PyroTyping and RFLP, hence, when PyroTyping profiles are the same, the isolates can be certainly clustered, and vice versa. Furthermore, direct analysis of patient specimens with PyroTyping will be set up, upon optimization of the protocol, in order to obtain enough double stranded high-quality DNA required for digestion.

The four typing methods performed in this study showed major discrepancies regarding the clustering or discrimination of the isolates in three case studies, mainly attributed to MIRU-VNTR. In one case, the two isolates were discriminated by RFLP, spoligotyping, and PyroTyping, but clustered by MIRU-VNTR. In the other two cases, the respective eight and two isolates were clustered by RFLP, spoligotyping, and PyroTyping, but one of the isolates in each case presented an additional band in RFLP, and the MIRU-VNTR patterns differed from the other isolates in three and seven loci, respectively. This may be explained by a higher discriminatory power of MIRU-VNTR. In addition, in other three case studies, although the isolates were clustered by the four typing methods, slight variations in the RFLP (one or two additional bands) and/or the MIRU-VNTR patterns (a different number of repeats in one loci) were detected. There has been some controversy on considering or not as being part of the same cluster isolates with those slightly different RFLP patterns^2, 10^, since RFLP profiles of isolates from a single source may differ in the presence or absence of at least one band^11^. In the same line, isolates with known epidemiological links and clustered by RFLP but showing single-locus variations in the MIRU-VNTR patterns have been reported^12, 13^. In the present study, we considered that these isolates were clustered because patients within case studies were epidemiologically related, isolates from related patients may present these minor changes^14^, the rate of transposition increases with the number of copies of IS*6110* ^2^, and the degree of clonality with MIRU-VNTR typing is high^15^. For those isolates that differed by one or two bands in the RFLP, the PyroTyping profiles were similar, and thus, the isolates were considered to be clustered by PyroTyping. It is of note that the height of some peaks in the pyrograms were slightly different, which may be the reflect of an additional sequence corresponding to the extra IS*6110* copy. However, the current accuracy of the method does not allow quantifying the exact number of nucleotides corresponding to the peak heights for subsequent comparison of isolates. In conclusion, the molecular relatedness of the isolates within case studies was supported by the different typing methods, although slightly more information was obtained by RFLP and MIRU-VNTR.

A drawback of IS*6110*-based genotyping methods is the reduced discriminatory power for low IS*6110* copy number isolates. RFLP clusters involving these isolates should be discriminated by another genotyping method not based on IS*6110*. Since PyroTyping is based on IS*6110*, it will not be informative for these isolates. However, in the present study, RFLP results regarding low IS*6110* copy number isolates were concordant with spoligotyping and MIRU-VNTR, and also with PyroTyping results. Variations of the method based on other repetitive elements present would be easily developed along the same lines reported here.

The PyroTyping assay can be performed in two days, the first day for digestion and ligation procedures, and the second one for PCR and pyrosequencing reaction. The overall procedure requires moderate training, especially for preparing the pyrosequencing reaction. In addition, PyroTyping requires specific equipment (pyrosequencer), but can be performed in 96-well plates, for high-throughput analysis, with a cost of reagents around 20USD per sample. Furthermore, PyroTyping profiles of the isolates within each case study were compared by the naked eye, therefore, the interpretation does not require a complex bioinformatics analysis, but for now it is restricted to a limited number of isolates with a suspected link. However, with a comprehensive software tool for storage and comparison of the PyroTyping profiles, a database for *M. tuberculosis* isolates from a geographical setting could be created, for clinical as well as for epidemiological purposes. In addition, upon optimization of the methodology, PyroTyping could be performed directly from clinical specimens. Moreover, since pyrosequencing has been used for detecting mutations associated with drug resistance^16, 17^, genotyping and detection of drug resistance could be performed in a combined assay, further increasing the clinical value of the technology for patient management. Lastly, the PyroTyping assay is potentially applicable for genotyping other bacterial species presenting polymorphism of insertion sequences, such as *Salmonella typhimurium* or *Staphylococcus aureus* ^18, 19^.

In conclusion, we have developed PyroTyping, a novel, rapid, and highly discriminatory assay that offers a promising alternative for *M. tuberculosis* genotyping for epidemiological studies in local and reference laboratories. The introduction of the touchdown PCR and pyrosequencing improved the performance over other methods analysing the variability on the flanking regions of the IS*6110* elements.

## Methods

### Clinical strains

During the period from 2006 to 2014, a total of 100 *M. tuberculosis* isolates were retrospectively selected. The isolates were received from different local clinical laboratories in Catalunya or were isolated in Hospital Universitari Germans Trias i Pujol. Almost one third of the patients were immigrants from different countries with high incidence of tuberculosis (Romania, Georgia, Morocco, Senegal, Mali, Gambia, Peru, Dominican Republic). Ninety-four of the 100 isolates corresponded to 29 molecular epidemiology case studies that were part of standard contact tracing investigations or suspected cases of laboratory cross-contamination (Table 1). The remaining six isolates were multidrug-resistant (MDR) *M. tuberculosis* isolates from patients from different countries (two patients from Morocco, two from Romania, one from Colombia, and one from Spain) for which there was not known or suspected link. All the isolates were subcultured on Löwenstein-Jensen (LJ) solid medium for at least four weeks or until colonies were well grown. All methods were carried out in accordance with relevant guidelines and regulations. This study was approved by the ethics committee in Institut Germans Trias i Pujol. Informed consent was obtained from all subjects.

### RFLP and spoligotyping

RFLP and spoligotyping were the routine molecular typing methods performed in Hospital Universitari Germans Trias i Pujol during the time of the study. DNA was extracted from isolates cultured on LJ following the cetyltrimethylammonium bromide (CTAB) protocol^20^. RFLP was performed as previously described^3^. Table 1 shows the molecular epidemiology investigation case studies, with the number of patients in each case study and the number of patient isolates clustered by RFLP.

Spoligotyping was performed using the spoligokit (Ocimum Biosolutions, Hyderabad, India) following the manufacturer’s instructions. The individual spoligotyping patterns were compared with those in the International Spoligotyping Database (SITVITWEB) of the Pasteur Institute of Guadeloupe, (http://www.pasteur-guadeloupe.fr:8081/SITVIT_ONLINE/). Spoligotyping International Types (SIT) were assigned according to spoligotype pattern signatures provided in SITVITWEB. Following the routine procedures, when RFLP and spoligotyping patterns were identical, isolates were considered to be clustered, whereas when patterns of RFLP and/or spoligotyping were different, isolates were considered to be unrelated. It is of note that RFLP patterns with a high number of bands that differed only in one or two bands were considered to be clustered^10, 14^.

### 24-loci MIRU-VNTR

Among the 100 *M. tuberculosis* isolates included in the study, 24-loci MIRU-VNTR was performed for the 86 isolates for which DNA was available. 24-loci MIRU-VNTR typing was performed using a quadriplex PCR provided in a commercial kit (Genoscreen, Lille, France) and processed with a 48-capillary ABI 3730 DNA Analyzer (Applied Biosystems, CA, USA). For MIRU-VNTR allele assignation GeneMapper software (Applied Biosystems, CA, USA) was used^21^. Genotyping was performed at the Laboratory of Molecular Epidemiology of Mycobacteria in Fundacion IIS Aragon, Zaragoza, Spain.

### PyroTyping

PyroTyping is a genotyping method based on the polymorphism of IS*6110* (Fig. 1). It consists of digestion of the *M. tuberculosis* genomic DNA with *Taq*I restriction enzyme, which cuts in a target (T^CGA) located within the IS*6110* and in a target located 5′ to the IS*6110*, dependent on insertion point; ligation of adaptors; touchdown PCR for amplification of the 5′ IS*6110*-flanking region of all the IS*6110* copies present in the genome; and simultaneous pyrosequencing of the amplified fragments. When two isolates share the same RFLP pattern, the IS*6110* copies are located in the same position in the genome, and thus, the sequence of the IS*6110*-5′ flanking regions would be identical. In this case, pyrosequencing profiles would be identical. On the contrary, when two isolates exhibit different RFLP patterns, pyrosequencing profiles would be different.

A first genomic digestion was performed in a final volume of 20 µl containing 5U *Tru*1I (*Mse*I) (Thermo Fisher Scientific, Waltham, MA, USA), 1X *Tru*1I buffer R (Thermo Fisher Scientific, Waltham, MA, USA), 0.1 mg/mL bovine serum albumin (BSA) (Hoffmann-La Roche, Basel, Switzerland), 0.5 mg/mL DNase-free RNase A (Hoffmann-La Roche, Basel, Switzerland), and 500 ng of CTAB-extracted genomic DNA. The Tru1I (MseI) digestion was carried out at 37 °C for at least 2 h. This digestion was performed in order to obtain smaller DNA fragments and facilitate the *Taq*I activity. A second digestion was performed by addition of 10U *Taq*I (Thermo Fisher Scientific, Waltham, MA, USA), and incubation at 65 °C for 3 h, and 80 °C for 2 min. Subsequently, a 24.6 µl ligation mix containing 40U of T4 DNA ligase (New England Biolabs, Ipswich, MA, USA), 2X T4 ligase buffer (Hoffmann-La Roche, Basel, Switzerland), and 0.2 µM of each adaptor (5′-CGGTCAGGACTCAT-3′, 5′-CGATGAGTCCTGAC-3′) (TIB MOLBIOL, Berlin, Germany) was added to the digestion product. Ligation was carried out at 12 °C for 17 h, and 65 °C for 10 min.

Touchdown PCR^22^ was performed in a final volume of 25 µl containing 1X HotStarTaq Master Mix (Qiagen, Venlo, The Netherlands), 1 µM each primer (forward 5′biotin-ATGAGTCCTGACCGA-3, reverse 5′-CTGACATGACCCCATCCTTT-3′) (TIB MOLBIOL, Berlin, Germany), 1 M betaine PCR reagent (Sigma-Aldrich, St. Louis, MO, USA), and 2.5 µl of ligation product. Touchdown PCR was carried out with the Veriti thermal cycler (Applied Biosystems, Foster City, CA, USA) and the following amplification conditions: 94 °C for 15 min; 10 cycles of 94 °C for 20 s, 66–56 °C for 30 s (temperature decreasing 1 °C every cycle from 66 °C to 56 °C), and 72 °C for 2 min; 20 cycles of 94 °C for 20 s, 56 °C for 30 s, and 72 °C for 2 min; and 72 °C for 7 min. Finally, pyrosequencing of the PCR product was performed using a PSQ 96MA and SQA software as recommended by the manufacturer (Qiagen, Venlo, The Netherlands). Briefly, the protocol consisted in the preparation of the single-stranded DNA with a vacuum preparation tool, annealing of the sequencing primer (5′-GGACATGCCGGGGCGGTT-3′) (TIB MOLBIOL, Berlin, Germany), and real-time pyrosequencing. In this protocol, the nucleotide dispensation order was 7x(ACTG), thus, the pyrograms presented 28 nucleotide peaks.

The result for each isolate consisted of a single pyrogram combining the simultaneous pyrosequencing of the 5′ flanking regions of all the IS*6110* copies present in the genome (Fig. 2). Therefore, the pyrogram corresponds to an artificial sequence obtained from the merged sequences of all the 5′ flanking regions. PyroTyping profiles from isolates from each case study were compared by the naked eye. When the same PyroTyping profiles were obtained, isolates were considered to be clustered, whereas when PyroTyping profiles were different, isolates were considered to be discriminated. Clustering or discrimination results obtained by PyroTyping were compared with those obtained by RFLP and spoligotyping. Spoligotyping and RFLP results were blinded to the researchers who interpreted PyroTyping results.

To assess the reproducibility of PyroTyping, three independent reactions were performed using DNA extracted from the *M. tuberculosis* reference strain H37Rv. In addition, to assess the limit of detection of the assay, six additional reactions were performed using ten-fold serial dilutions of DNA extracted from H37Rv (100ng to 0.001ng).

### Data Availability

The datasets generated during and/or analysed during the current study are available from the corresponding author on reasonable request.

## Electronic supplementary material

Supplementary Information
