# Supplementary material for: PyroTyping, a novel pyrosequencing-based assay for Mycobacterium tuberculosis genotyping
Source: Sci Rep. 2017 Jul 28;7:6777. doi: 10.1038/s41598-017-06760-5 (PMC5533701; doi:10.1038/s41598-017-06760-5)
Supplement: Supplementary file 1 — Supplementary Information [file 41598_2017_6760_MOESM1_ESM.pdf]

Title: PyroTyping, a novel pyrosequencing-based assay for *Mycobacterium tuberculosis* genotyping

Authors:

Molina-Moya B[a,c,#], Lacoma A[a,c,#], Garcia-Sierra N[a], Blanco S[a,c], Haba L[a], Samper S[c,d,e], Ruiz-Manzano J[b,c], Prat C[a,c,$], Arnold C [f,$], Domínguez J[a,c,$,*].

[a] Servei de Microbiologia i [b] Servei de Pneumologia, Hospital Universitari Germans Trias i Pujol. Institut d'Investigació Germans Trias i Pujol. Universitat Autònoma de Barcelona. Carretera del Canyet s/n, 08916 Badalona, Spain. [c] CIBER Enfermedades Respiratorias (CIBERES), Instituto de Salud Carlos III, Spain. [d] Instituto Aragonés de Ciencias de la Salud, Zaragoza, Spain. [e] Fundación Instituto de Investigacion Sanitaria de Aragón, Zaragoza, Spain. [f] Genomic Services and Development Unit, Public Health England, 61 Colindale Avenue, London, United Kingdom

[#] These authors contributed equally to this work.

[$] Co-senior authors.

Corresponding author:

Jose Domínguez

Edifici Laboratoris de Recerca.

Institut d'Investigació Germans Trias i Pujol.

Carretera del Canyet, Camí de les Escoles, s/n.

08916 Badalona, Barcelona, Spain

Phone: +34 93 497 86 97

E-mail: jadominguez@igtp.cat

SUPPLEMENTARY INFORMATION

Spoligotyping and 24-loci MIRU-VNTR results for the 100 *M. tuberculosis* isolates included in the study.

Case nr.<br>Isolate nr	Type of investigation	Spoligotype	Spoligotype43	SIT	Clade	Family	MIRU-VNTR 24 loci
1.1	Contact tracing - family members	□□□□□□□□□□□□□□□□□□□□□□□□■■■■■■■■□□□□■■■■■■■	000000007760771	4	Orphan	Orphan	124264332214126153322522
1.2	Contact tracing - family members	□□□□□□□□□□□□□□□□□□□□□□□□■■■■■■■■□□□□■■■■■■■	000000007760771	4	Orphan	Orphan	124264332214126153322522
1.3	Contact tracing - family members	□□□□□□□□□□□□□□□□□□□□□□□□■■■■■■■■□□□□■■■■■■■	000000007760771	4	Orphan	Orphan	124264332214126153322522
2.1	Cross-contamination	■□■■■■■■■■■■■■■■■■■■■■■■■■■■■■■■□□□□■■■■■■■	577777777760771	334	T1	T	Not performed
2.2	Cross-contamination	■□■■■■■■■■■■■■■■■■■■■■■■■■■■■■■■□□□□■■■■■■■	577777777760771	334	T1	T	Not performed
2.3	Cross-contamination	■□■■■■■■■■■■■■■■■■■■■■■■■■■■■■■■□□□□■■■■■■■	577777777760771	334	T1	T	Not performed
3.1	Cross-contamination	■■■■■■■■□■□■□■□■■■□■□■□□□■□□□□□□□□□□□□□□□□□	776527242000000	Orphan	Orphan	Orphan	Not performed
3.2	Cross-contamination	■■■■■■■■■■■■■■■■■■■□□■■■■■■■□□□□□□□□□□□□□□■	777777477400001	160	Orphan	Orphan	Not performed
3.3	Cross-contamination	■■■■■■■■■■■■■■■■■■■□□■■■■■■■□□□□□□□□□□□□□□■	777777477400001	160	Orphan	Orphan	Not performed
4.1	Cross-contamination	■■□□■■■■■■■■■■■■■■■■■■■■■■■■■■□■□□□□■■■■■■■	637777777720771	849	H3	Haarlem	Not performed
4.2	Cross-contamination	■■■■■■■■□□■■■■■■■■□□□□□□□■■■■■■■□□□□■■■■■■■	776377003760771	Orphan	Orphan	Orphan	Not performed
4.3	Cross-contamination	■■■■■■■■■■■■□□■■■■■■□□□□■■■■■■■■□□□□■■■■■■■	777717607760771	216	LAM5	LAM	Not performed
4.4	Cross-contamination	■■■■■■■■■■■■□□■■■■■■□□□□■■■■■■■■□□□□■■■■■■■	777717607760771	216	LAM5	LAM	Not performed
4.5	Cross-contamination	■■■■■■■■■■■■□□■■■■■■□□□□■■■■■■■■□□□□■■■■■■■	777717607760771	216	LAM5	LAM	Not performed
4.6	Cross-contamination	■■■■■■■■■■■■□□■■■■■■□□□□■■■■■■■■□□□□■■■■■■■	777717607760771	216	LAM5	LAM	Not performed
5.1	Cross-contamination	■■■■■■■■■■■■■■□■■■■□□■■■■■■■□□□□□□□□□□□□□□■	777767477400001	Orphan	Orphan	Orphan	Not performed
5.2	Cross-contamination	■■■■■■■■■■■■■■□■■■■□□■■■■■■■□□□□□□□□□□□□□□■	777767477400001	Orphan	Orphan	Orphan	22324222253442515-333-32
6.1	Contact tracing - family members	■■□■■■■■■■■■□■■■■■■■□□□□■■■■■■■■□□□□■■■■■■■	677737607760771	17	LAM2	LAM	234214232224125163333522
6.2	Contact tracing - family members	■■□■■■■■■■■■□■■■■■■■□□□□■■■■■■■■□□□□■■■■■■■	677737607760771	17	LAM2	LAM	234214232224125163333522
7.1	Contact tracing - spatio-temporal proximity	■■■■■■□□□■■■■■■■■■■■■■■■■■□■■■■■■■■■■■■□■■■■	770777775777671	Orphan	Orphan	Orphan	215217242473444242354432
7.2	Contact tracing - spatio-temporal proximity	■■■■■■□□□■■■■■■■■■■■■■■■■■□■■■■■■■■■■■■□■■■■	770777775777671	Orphan	Orphan	Orphan	215217242473444242354432
7.3	Contact tracing - spatio-temporal proximity	■■■■■■■■■■■■■■■■■■■■□□□□■■■■■■■■□□□□■■■■■■■	777777607760771	42	LAM9	LAM	134264332224126143332612
7.4	Contact tracing - spatio-temporal proximity	■■■■■■■■■■■■□■■■■■■■■■■■■■■■□□□■□□□□■■■■■■■	777737777420771	35	H3	Haarlem	244229232234415113333632
7.5	Contact tracing - spatio-temporal proximity	■■■■■■□□□■■■■■■■■■■■■■■■■■□■■■■■■■■■■■■□■■■■	770777775777671	Orphan	Orphan	Orphan	215217242473444242354332
7.6	Contact tracing - spatio-temporal proximity	■■■□□□□■■■■■■■■■■■□■■■□□□□□□□□□□□□■■□□■■■■■	703777340003171	Orphan	Orphan	Orphan	232236442--4225153353643
7.7	Contact tracing - spatio-temporal proximity	■■■■■■□□□■■■■■■■■■■■■■■■■■□■■■■■■■■■■■■□■■■■	770777775777671	Orphan	Orphan	Orphan	215217242473444242354432
7.8	Contact tracing - spatio-temporal proximity	■■■■■■■■■■■■□■■■■■■■■■■■■■■■□□□■□□□□■■■■■■■	777737777420771	35	H3	Haarlem	244229232234415113333632
7.9	Contact tracing - spatio-temporal proximity	■■■■■■■■■■■■■■■■■■■■■■■■■■■■■■■■□□□□■■■■■■■	777777777760771	53	T1	T	224223322524225143336522
7.10	Contact tracing - spatio-temporal proximity	■■■■■■■■■■■■■■■■■■■■□□□□■■■■■■■■□□□□■■■■■■■	777777607760771	42	LAM9	LAM	134264332224126143332612
7.11	Contact tracing - spatio-temporal proximity	■■■■■■■■■■■■■■■■■■■■□■■■■■■■■■■■□□□□■■■■■■■	777777677760771	291	T1	T	224223522224225153334522
7.12	Contact tracing - spatio-temporal proximity	■■■■■■■■■■■■■■■■■■■■□■■■■■■■■■■■□□□□■■■■■■■	777777677760771	291	T1	T	224223522224225153334522
7.13	Contact tracing - spatio-temporal proximity	■■■■■■■■■■■■■■■■■■■■□□□□■■■■■■■■□□□□■■■■■■■	777777607760771	42	LAM9	LAM	24421422242411613-532-22
7.14	Contact tracing - spatio-temporal proximity	■■■■■■■■■■■■■■■■■■■□■■□■■■■■■■■■■□□□□■■■■■■■	777777557760771	58	T5-Madrid2	T	224243322334226133333522
7.15	Contact tracing - spatio-temporal proximity	■■■■■■□□□■■■■■■■■■■■■□□□■■■■■■■■■■■■■■□■■■■	770777707777671	326	AFRI_1	AFRI	235225462463444243353632

Case nr. Isolate nr	Type of investigation	Spoligotype	Spoligotype43	SIT	Clade	Family	MIRU-VNTR 24 loci
8.1	Cross-contamination	■■□■■■■■■■■■■■■■■■■■□□□□□□□■■■■■□□□□■■■■■■■	677777600760771	Orphan	Orphan	Orphan	244213232214126163222422
8.2	Cross-contamination	■□□□■■■■□□□■■■■■■■■■□□□□■■■■■■■■□□□□■■■■■■■	436177607760771	Orphan	Orphan	Orphan	222234222424226153131722
8.3	Cross-contamination	■■□■■■■■■■■■■■■■■■■■□□□□□□□■■■■■□□□□■■■■■■■	677777600760771	Orphan	Orphan	Orphan	244213232214126163222422
9.1	Contact tracing - family members	■■■■■□□□□□□□□□□□□□□□□□□□□■■■■■■■■□□□□■■■■■■■	760000007760771	1574	LAM3	LAM	2442453224242241341-2632
9.2	Contact tracing - family members	■■■■■□□□□□□□□□□□□□□□□□□□□■■■■■■■■□□□□■■■■■■■	760000007760771	1574	LAM3	LAM	244234322424224133131622
9.3	Contact tracing - family members	■■■■■□□□□□□□□□□□□□□□□□□□□■■■■■■■■□□□□■■■■■■■	760000007760771	1574	LAM3	LAM	244234322424224133131622
10.1	Contact tracing - roommate	■■■■■■■■■■■■■■■■■■■■■■■■■■■■■■□■□□□□■■■■■■■	777777777720771	50	H3	Haarlem	223345331432425153324732
10.2	Contact tracing - roommate	■■■■■■■■■■■■■■■■■■■■■■■■■■■■■■□■□□□□■■■■■■■	777777777720771	50	H3	Haarlem	223345331432425153324732
10.3	Contact tracing - roommate	■■■■■■□□■■■■□■■■■■■■■■■■■■■■■■■□□□□■■■■■■■	771737777760771	Orphan	Orphan	Orphan	224223422424227154333522
11.1	Cross-contamination	■■■■■■■■■■■■■■■■■■■■□□□□■■■■■■■■□□□□■■■□■■■	777777607760731	60	LAM4	LAM	214213232424116143532422
11.2	Cross-contamination	■■■■■■■■■■■■■■■■■■■■□□□□■■■■■■■■□□□□■■■□■■■	777777607760731	60	LAM4	LAM	214213232424116143532422
11.3	Cross-contamination	■■■■■■■■■■■■■■■■■■■■□□□□■■■■■■■■□□□□■■■□■■■	777777607760731	60	LAM4	LAM	214213232424116143532422
12.1	Contact tracing - family members	■■■■■□□□□□□□□□□□□□□□□□□□□■■■□□□■■□□□□■■■■■■■	760000007060771	Orphan	Orphan	Orphan	234244322424226133131322
12.3	Contact tracing - family members	■■■■■□□□□□□□□□□□□□□□□□□□□■■■□□□■■□□□□■■■■■■■	760000007060771	Orphan	Orphan	Orphan	234244322424226133131322
13.1	Cross-contamination	■■■■■■■□□□■□■□□□■□■■■■■■■■■■■■■■■■■■■■■■■■■	774242777777771	Orphan	Orphan	Orphan	213235332734425153333532
13.2	Cross-contamination	■■■■■■■□□□■□■□□□■□■■■■■■■■■■■■■■■■■■■■■■■■■	774242777777771	Orphan	Orphan	Orphan	213235332734425153333532
14.1	Contact tracing - friends	■■■■■■■■■■■■■■■■■■■■□□□□■■■■■■■■□□□□■■■■■■■	777777607760771	42	LAM9	LAM	244213232224116143532822
14.2	Contact tracing - friends	■■■■■■■■■■■■■■■■■■■□□■■■■■■■□□□□□□□□□□□□□□■	777777477400001	160	Orphan	Orphan	223232322534425153333432
15.1	Contact tracing - family members	■■■■■■■■■■■■■■■■■■■■■■■■■■■■□■□■□□□□■■■■■■■	777777777520771	746	H3	Haarlem	223236322634425153333332
15.2	Contact tracing - family members	■■■■■■■■■■■■■■■■■■■■■■■■■■■■□■□■□□□□■■■■■■■	777777777520771	746	H3	Haarlem	223236322634425153333332
16.1	Contact tracing - roommate	■■■■■■■■■■■■■■■■■■■■■■■■■■■□□□□□□□□□□■■■■■■	777777777000371	775	Orphan	Orphan	223242322234226143333522
16.2	Contact tracing - roommate	■■■■■■■■■■■■■■■■■■■■■■■■■■■□□□□□□□□□□■■■■■■	777777777000371	775	Orphan	Orphan	223242322234226143333522
17.1	Contact tracing - spatio-temporal proximity	■■■■■■■■■■■■■■■■■■■■■■■■■■■■■■■■□□□□■■■■■■■	777777777760771	53	T1	T	223223422524225163331422
17.2	Contact tracing - spatio-temporal proximity	■■■■■■■■■■■■■■■■■■■■■■■■■■■■■■■■□□□□■■■■■■■	777777777760771	53	T1	T	226223422524225163331422
17.3	Contact tracing - spatio-temporal proximity	■■■■■■■■■■■■■■■■■■■■■■■■■■■■■■■■□□□□■■■■■■■	777777777760771	53	T1	T	22f223422524225163331422
17.4	Contact tracing - spatio-temporal proximity	■■■■■■■■■■■■■■■■■■■■■■■■■■■■■■■■□□□□■■■■■■■	777777777760771	53	T1	T	226223422524225163331422
17.5	Contact tracing - spatio-temporal proximity	■■■■■■■■■■■■■■■■■■■■■■■■■■■■■■■■□□□□■■■■■■■	777777777760771	53	T1	T	22f223422524225163331422
18.1	Contact tracing - spatio-temporal proximity	■■■■■■■■■■■■□■■■■■■■■■■■□□□□□□■□□□□■■■■■■■	777737774020771	218	H1	Haarlem	223235332734425153333412
18.2	Contact tracing - spatio-temporal proximity	■■■■■■■■■■■■■■■■■■■■□□□□■■■■■■■■□□□□■■■■■□■	777777607760761	1074	LAM9	LAM	245254332424224143333822
19.1	Contact tracing - colleagues at work	■■■■■■■■■■■■■■■■■■■□■■□■■■■■■■■■■□□□□■■■■■■■	777777557760771	58	T5-Madrid2	T	252244232424226153131722
19.2	Contact tracing - colleagues at work	■■■■■■■■□□□■■■■■■■■■■□□□□■■■■■■■■□□□□■■■■■■■	776177607760771	33	LAM3	LAM	252244232424226153131722
20.1	Contact tracing - roommate	■■■■■■■■■■■■■■■■■■■■■■■■■■■■■■■■□□□□■■■■■■■	777777777760771	53	T1	T	224243122434225153335522
20.2	Contact tracing - roommate	■■■■■■■■■■■■■■■■■■■■□□□□■■■■■■■■□□□□■■■■■■■	777777607760771	42	LAM9	LAM	242224322224226163345622
21.1	Contact tracing - nursery school	■■■■■■■□□□□■□■■■■■■■□□□□□□□□□□□■□□□□■■■■■■■	774137600020771	1538	H3	Haarlem	244224332124226153131622
21.2	Contact tracing - nursery school	■■■■■■■□□□□■□■■■■■■■□□□□□□□□□□□■□□□□■■■■■■■	774137600020771	1538	H3	Haarlem	244224332124226153131622

Case nr. Isolate nr	Type of investigation	Spoligotype	Spoligotype43	SIT	Clade	Family	MIRU-VNTR 24 loci
22.1	Contact tracing - family members	■■■■■■■■■□■□■□□□□■■■■■■■■■■■□■■■□□□□■■■■■■■	777241777560771	Orphan	Orphan	Orphan	224223322424225154343522
22.2	Contact tracing - family members	■■■■■■■■■□■□■□□□□■■■■■■■■■■■□■■■□□□□■■■■■■■	777241777560771	Orphan	Orphan	Orphan	224223322424225154343522
22.3	Contact tracing - family members	■■■■■■■■■□■□■□□□□■■■■■■■■■■■□■■■□□□□■■■■■■■	777241777560771	Orphan	Orphan	Orphan	224223322424225154343522
22.4	Contact tracing - family members	■■■■■■■■■□■□■□□□□■■■■■■■■■■■□■■■□□□□■■■■■■■	777241777560771	Orphan	Orphan	Orphan	224223322424225154343522
23.1	Contact tracing - family members	■■■■■■■■■■■■■■■■■■■□■■□■■■■■■■■■□□□□■■■■■■■	777777557760771	58	T5-Madrid2	T	224233322334226153432522
23.2	Contact tracing - family members	■■■■■■■■■■■■■■■■■■■□■■□■■■■■■■■■□□□□■■■■■■■	777777557760771	58	T5-Madrid2	T	224233322334226153432522
23.3	Contact tracing - family members	■■■■■■■■■■■■■■■■■■■□■■□■■■■■■■■■□□□□■■■■■■■	777777557760771	58	T5-Madrid2	T	224233322334226153432522
24.1	Contact tracing - colleagues at work	■■■■■■■■■■■■■■■■■■■■■■■■■■■■■■□■□□□□■■■■■■■	777777777720771	50	H3	Haarlem	223235322534425143333732
24.2	Contact tracing - colleagues at work	■■■■■■■■□□□■■■■■■■■■□□□□■■■■■■■■□□□□■■■■■■■	776177607760771	33	LAM3	LAM	244214322424226153131722
25.1	Contact tracing - family members	□■■■□■■■■■■■■■■■■■■■■■■■■■■■■■■■□□□□■■■■■■■	357777777760771	Orphan	Orphan	Orphan	224223322224224152333422
25.2	Contact tracing - family members	□■■■□■■■■■■■■■■■■■■■■■■■■■■■■■■■□□□□■■■■■■■	357777777760771	Orphan	Orphan	Orphan	224223322224224152333422
26.1	Contact tracing - colleagues at work	■■■■■■■■■■■■■■■■■■■□□□□□■□□■■■■■□□□□■■■■■■■	777777404760771	41	LAM7-TUR	LAM	244125132234425113333732
26.2	Contact tracing - colleagues at work	■■■■■■■■■■■■■■■■■■■□□□□□■□□■■■■■□□□□■■■■■■■	777777404760771	41	LAM7-TUR	LAM	244125132234425113333732
27.1	Contact tracing - family members	■■■■■■■■■■■■□■■■■■■■■■■■■■■■■■■■□□□□■■■■■■■	777737777760771	37	T3	T	223223322324225153335522
27.2	Contact tracing - family members	■■■■■■■■■■■■□■■■■■■■■■■■■■■■■■■■□□□□■■■■■■■	777737777760771	37	T3	T	Not performed
28.1	Contact tracing - spatio-temporal proximity	■■■■■■■■□□□■■□□□□□□□□□□□□□□□□□□□□□□□□□□■■■■	776140000000071	Orphan	Orphan	Orphan	224244322324226133131622
28.2	Contact tracing - spatio-temporal proximity	■■■■■■■■□□□■■□□□□□□□□□□□□□□□□□□□□□□□□□□■■■■	776140000000071	Orphan	Orphan	Orphan	224244322324226133131622
28.3	Contact tracing - spatio-temporal proximity	■■■■■■■■□□□■■□□□□□□□□□□□□□□□□□□□□□□□□□□■■■■	776140000000071	Orphan	Orphan	Orphan	224244322324226133131622
28.4	Contact tracing - spatio-temporal proximity	■■■■■■■■□□□■■□□□□□□□□□□□□□□□□□□□□□□□□□□■■■■	776140000000071	Orphan	Orphan	Orphan	224244322324226133131622
28.5	Contact tracing - spatio-temporal proximity	■■■■■■■■□□□■■□□□□□□□□□□□□□□□□□□□□□□□□□□■■■■	776140000000071	Orphan	Orphan	Orphan	224244322324226133131622
28.6	Contact tracing - spatio-temporal proximity	■■■■■■■■□□□■■□□□□□□□□□□□□□□□□□□□□□□□□□□■■■■	776140000000071	Orphan	Orphan	Orphan	224244322324226133131622
28.7	Contact tracing - spatio-temporal proximity	■■■■■■■■□□□■■□□□□□□□□□□□□□□□□□□□□□□□□□□■■■■	776140000000071	Orphan	Orphan	Orphan	224244322324226133131622
28.8	Contact tracing - spatio-temporal proximity	■■■■■■■■□□□■■□□□□□□□□□□□□□□□□□□□□□□□□□□■■■■	776140000000071	Orphan	Orphan	Orphan	224224322524226153131622
29.1	Contact tracing - spatio-temporal proximity	■■■■■■■■■■■■■■■■■■■■■■■■■■■■■■■■□□□□■■■■■■■	777777777760771	53	T1	T	224244322324225133333422
29.2	Contact tracing - spatio-temporal proximity	■■■■■■■■■■■■■■■■■■■■■■■■■■■■■■■■□□□□■■■■■■■	777777777760771	53	T1	T	224213322234225153334522
MDR.1	Unrelated multidrug-resistant isolate	■■■■■■■■■■■■■□■■■■■■■■■■■□■□□□□■□□□□■■■■■■■	777757775020771	Orphan	Orphan	Orphan	223225321432423153333522
MDR.2	Unrelated multidrug-resistant isolate	□□□□□□□□□□□□□□□□□□□□□□□□□□□□□□□□□□■■■■■■■■■	000000000003771	1	Beijing	Beijing	244233352644425153353624
MDR.3	Unrelated multidrug-resistant isolate	■■■■■■□■□□■■□■□□□□■■■■■■■■■■■■■■■□□□■■■■■■■	772320777770771	Orphan	Orphan	Orphan	252236342244225173353843
MDR.4	Unrelated multidrug-resistant isolate	■■■■■■■■■■■■■■■■■■■■■■■■■■■■■■■■□□□□■■■■■■■	777777777760771	53	T1	T	334223322334225163333622
MDR.5	Unrelated multidrug-resistant isolate	■■■■■■■■□□□■■■□□□□□□□□□□□□□□□□□□□□□□□□□■■■■	776160000000071	105	Orphan	Orphan	224244322224226133131622
MDR.6	Unrelated multidrug-resistant isolate	■■■□□□□□□□□□□□□□□■■■■■■■■□□□□□□■□□□□■■■■■■■	700001774020771	Orphan	Orphan	Orphan	224243122434225153543522
f: >15 repeats							
